# Oversummering juvenile and adult Semipalmated sandpipers in Perú gain enough survival to compensate for foregone breeding opportunity

**DOI:** 10.1186/s40462-020-00226-6

**Published:** 2020-10-27

**Authors:** Eveling A. Tavera, Glenn E. Stauffer, David B. Lank, Ronald C. Ydenberg

**Affiliations:** 1 Centre for Wildlife Ecology, Department of Biological Sciences, Simon Fraser University, 8888 University Drive, Burnaby, British Columbia V5A 1S6 Canada; 2 Centro de Ornitología y Biodiversidad – CORBIDI, Santa Rita 105, Of. 202, Huertos de San Antonio, Surco, Lima 33, Lima, Peru; 3 Present address: Centre for Wildlife Ecology, Department of Biological Sciences, Simon Fraser University, 8888 University Dr., Burnaby, BC V5C2G2 Canada; 4 Wisconsin Department of Natural Resources, 107 Sutliff Ave, Rhinelander, WI 54501 USA

**Keywords:** *Calidris pusilla*, Oversummering, Survivorship, Multi-state mark-recapture model, Migratory strategy, Distance-dependant, Shorebirds, Paracas, Perú

## Abstract

**Background:**

Age at maturity and the timing of first breeding are important life history traits. Most small shorebird species mature and breed as ‘yearlings’, but have lower reproductive success than adults. In some species, yearlings may defer northward migration and remain in non-breeding regions (‘oversummering’) until they reach 2 years of age. Some adults also oversummer. Oversummering would be favoured by natural selection if survival were as a result raised sufficiently to compensate for the missed breeding opportunity. Several thousand Semipalmated Sandpipers (*Calidris pusilla*) spend the non-breeding period at Paracas, Perú, including individuals with long bills (likely from eastern Arctic breeding populations ~ 8000 km distant) and short bills (likely from western Arctic breeding populations, up to 11,000 km distant), with short-billed birds more likely to oversummer. We tested the prediction that oversummering birds have higher survival than migrants, and that the magnitude of this higher survival for oversummering birds is enough to compensate for their lost breeding season.

**Methods:**

We used a Multi-State Mark-Recapture model based on 5 years of encounter data (*n* = 1963 marked birds, and 3229 resightings) obtained year-round at Paracas, Perú, to estimate seasonal (i.e. breeding and non-breeding) survivorship for migrant and oversummering birds. We calculated the magnitude of the oversummering survival advantage required to compensate, for both yearlings and adults, based on published measures of annual survival and reproductive success. Using bill length as a proxy for migration distance, we investigated whether migratory survival is distance-dependent.

**Results:**

We estimate that 28% of yearlings and 19% of adults oversummer. Survival is higher for oversummering birds than for migrants, and the oversummering survival advantage is greater for adults (0.215) than for yearlings (0.140). The theoretical thresholds predicted by the size of the missed reproductive opportunity are 0.240 for adults and 0.134 for yearlings. Migratory survival decreases and the oversummering rate increases with migration distance, as assessed by culmen length.

**Conclusions:**

Our results support the life history hypothesis that oversummering raises survival enough to compensate for the loss of a breeding opportunity. Greater migration distance lowers survival and increases the probability of oversummering.

## Background

Life history theory predicts that natural selection acts on the age of maturity through its effects on survivorship and reproductive success [1]. The age of first breeding can have a substantial effect on population growth rate, and cases in which individuals forgo early breeding opportunities are therefore of intrinsic interest [2]. Among small shorebird species, most individuals attempt to breed in their first year of life, e.g. Dunlin *Calidris alpina* [3], Temminck’s Stint *Calidris temminckii* [4], Least Sandpiper *Calidris minutilla* [5] and Semipalmated Sandpiper *Calidris pusilla* [6]. As in birds in general, first year breeders have lower reproductive success than older individuals [7]. In Semipalmated and Western Sandpipers *Calidris mauri* young breeders have later hatch dates, smaller egg sizes, lower nesting success and lower fecundity than adults [6] (Kwon E, et al. Age-specific fecundity and population dynamics of Western Sandpipers Calidris mauri. In prep.). The lower reproductive payoffs for young birds help to favor delayed breeding, and factors that increase the risk or cost of migration further raise the survival advantage of delayed breeding.

‘Oversummering’ is a term used to describe when individuals in a typically migratory shorebird species defer migration and remain on the non-breeding grounds during the breeding season [8]. (As in most literature, ‘breeding season’ here refers to the boreal spring and summer.) Oversummering has been variously attributed to sexual immaturity [9, 10]; helminthic infestation [8]; sterility, injuries or illness [11]; less efficient foraging [12]; flight cost on primary wear [13]; behavioral adaptations to distance-dependent costs [14–16] and poor success in the first breeding attempt [17]. Summers et al. [17] found that among five species of shorebirds with groups spending the non-breeding season in Britain or South Africa, a large proportion of South African birds showed no preparation for migration (molt to breeding plumage and mass gain). They inferred that these birds oversummered, attributing this to distance-dependant migration risk. Migration distance has also been used as a factor to explain intraspecific differences in the age of first breeding within Western Sandpipers and Sanderlings *Calidris alba*. Juveniles at more southerly non-breeding areas, further from arctic breeding grounds, neither molt into breeding plumage nor migrate northward, while those at more northerly locations do so [18–23].

In this study we test the hypothesis that oversummering provides a survival advantage over migration. We predict that oversummering enhances survivorship of those individuals doing so by enough to offset the expected fitness cost of their foregone breeding opportunity. We also evaluate whether migratory survival falls with distance as previous investigators have suggested, and if so, whether oversummering is as predicted more prevalent when migrations are longer.

Semipalmated Sandpipers perform an annual return migration between South American non-breeding regions and Arctic breeding areas ranging from Alaska eastward across the Canadian tundra [20, 24]. Adults undergo a full molt after southward migration, upon (or just before) returning to non-breeding sites. Juveniles migrate a full month later than adults and do not molt, though some later undertake a partial wing molt (replacing 1–6 primaries; termed ‘partial post-juvenal wing molt, or ‘PPW’) during the pre-migratory period (January – March). At our study area at Paracas, Perú, at the southern edge of the non-breeding range, many young birds oversummer [18], as do some adults.

This migratory dichotomy provides an opportunity to compare the survival of oversummering and migrant birds. To do so we develop a multi-state mark-capture-resighting (MSMR) model with two age classes (juveniles/yearlings, adults) and two migration strategies (oversummer, migrate). We predict that oversummering birds have higher survival than migrants during the breeding season (April – September). Further, since adults have a greater probability of breeding successfully than juveniles, those that oversummer should gain more in terms of survivorship than young birds by doing so. Finally, among birds that do migrate, we investigate whether migratory survival is distance dependent, using bill length as a proxy for migration distance (see below). If so, birds presumed to be from western Arctic breeding populations (short bills and long migrations) should be more likely to oversummer than those presumed to be from eastern Arctic breeding populations (long bills and shorter migrations).

## Methods

### Study site

**Fig. 1:**
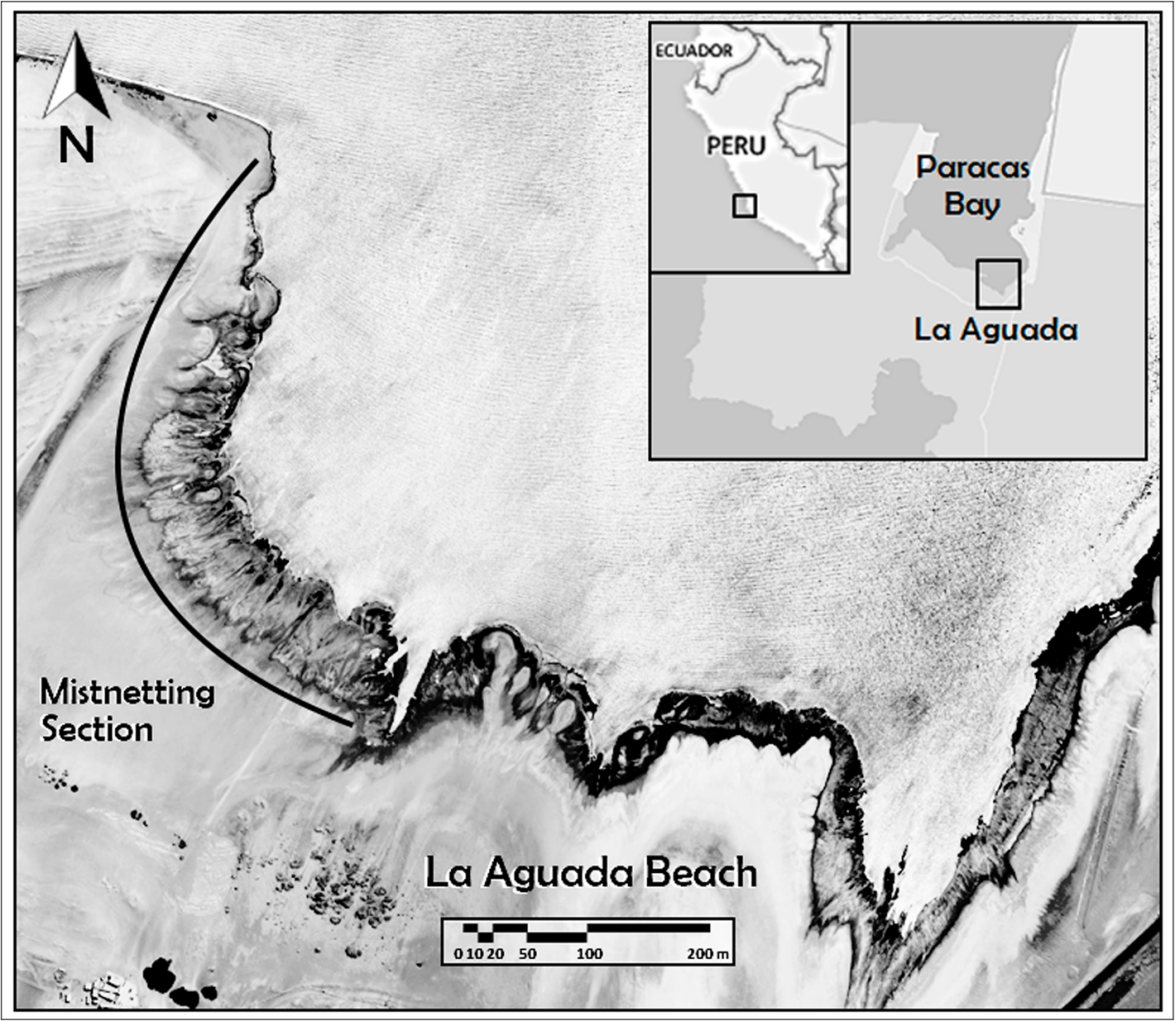
Location of the study site, La Aguada Beach at Paracas National Reserve, Department of Ica, Perú

We captured, marked, released, and resighted Semipalmated Sandpipers between October 2014 and March 2019, at the Paracas National Reserve in Perú, a natural protected area located in the department of Ica, 250 km south of Lima city (Fig. 1). The work was conducted on La Aguada beach (13° 51′ 35′′S, 76° 16′ 16′′ W), an intertidal mudflat ~ 2 km long and surrounded by coastal desert. The broad near-shore section of the mudflat has no vegetation and is inundated only on the highest monthly tides. The intertidal mudflat follows the fringe of the bay, is ~ 50 m wide, and is inundated twice daily by tides of ~ 1.5 m in height.

### Capture, marking and resighting

Fieldwork was conducted during both the non-breeding season (October to March; termed ‘winter’) and the migration/breeding season (April to September; termed ‘summer’). During non-breeding seasons, we conducted seven-day capture-resighting ‘field campaigns’ during the new moon phase of each month. Shorebirds were captured at night (2000 – 0600 h) with mist-nets, beginning 3 h after the evening high tide and ending 3 h before the subsequent high tide. Captured birds were marked on the right tarsus with an incoloy metal band obtained from the CORBIDI Bird-Banding Program (the Peruvian bird-banding scheme). A three-character-coded yellow flag was placed on the left tibia (e.g. 3AT), following the Pan-American Shorebird Program protocol [25], to identify individuals and enable resightings. Each morning, 3 persons each spent 3 h (0600 - 0900 h) surveying the entire study area, locating and identifying (by telescope) marked individuals. During breeding seasons we did no mist-netting, but carried out a 5-day resighting-only field campaign each month. All capture, handling and marking methods were approved by regulatory committees for animal welfare and permitting agencies for wildlife research.

Upon initial capture, birds were assigned to an age category based on plumage characteristics and date. Young-of-the-year are first seen in Paracas in September, and are considered ‘juveniles’ until April 1 of the following year (~ 10 months of age) when they by definition become ‘yearlings’. They are recognizable by plumage, particularly the retained juvenile-type inner greater coverts [20, 26]. Field campaigns during the summer months are ‘resighting only’ and by the time mist-netting resumes in October of each year, all yearlings have completed molt into adult plumage and are easily distinguished from newly-arrived juveniles. Adult plumage is distinct, recognizable by the shape and coloration of newly molted primaries [20, 26].

Culmen length was measured using a dial caliper (mm). Semipalmated Sandpipers have a cline in bill length across their breeding range, with average bill length shorter in western breeding populations [24, 27]. The distribution of bill lengths at Paracas encompasses the full range, and is slightly left-skewed (towards shorter bills [18];). These data suggest that Semipalmated Sandpipers at Paracas include birds from western (~ 11,000 km distant on a great circle route) as well as eastern Arctic breeding populations (~ 8000 km).

### Multi-state model structure

**Fig. 2:**
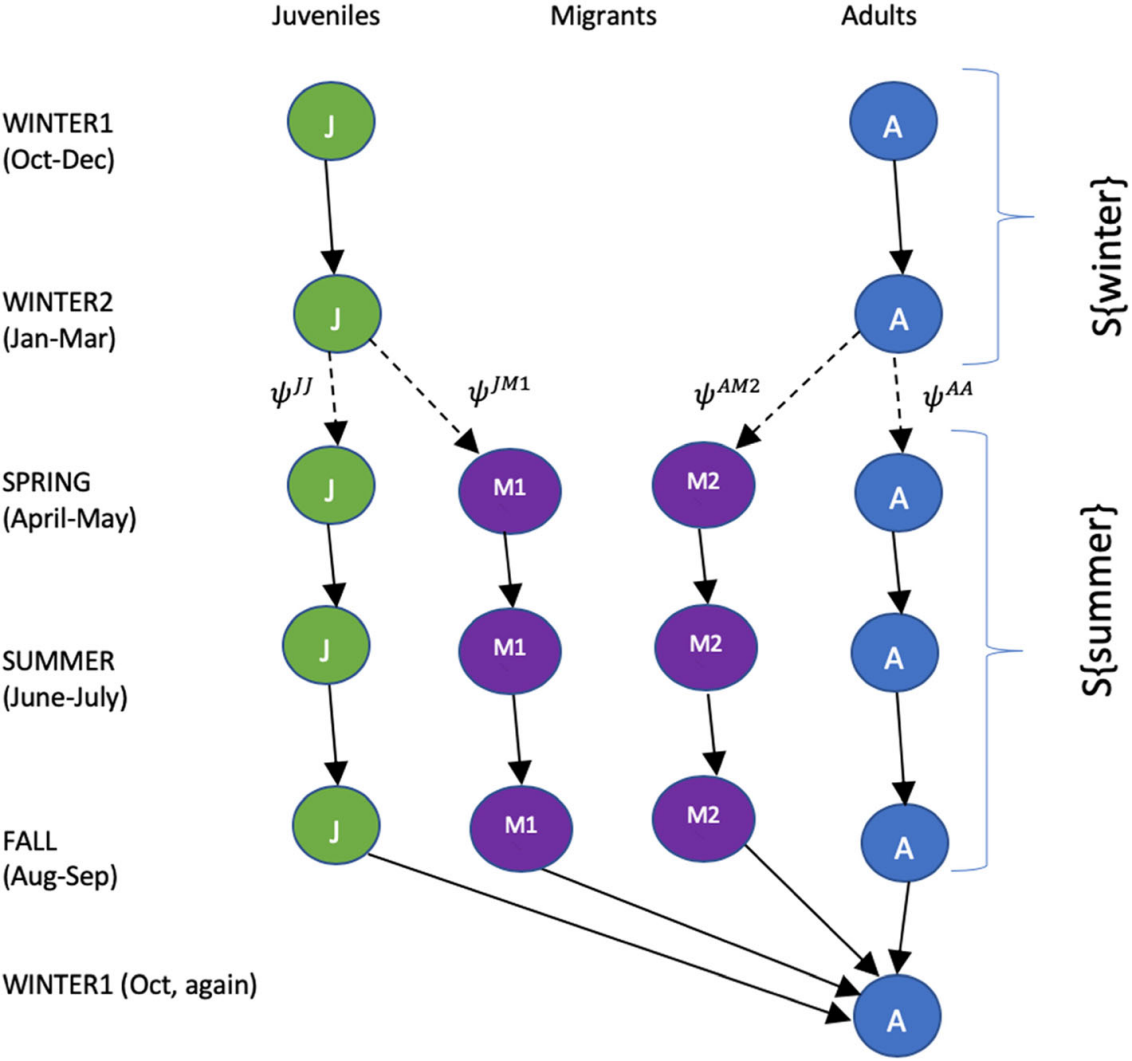
State transitions for Semipalmated Sandpipers at Paracas, Perú. Solid arrows denote compulsory transitions, and dashed transitions denote probalistic transitions estimated by the multi-state mark-recapture (MSMR) model. Sampling occasions (Winter 1, Winter2, Spring, Summer and Fall) are not of equal length The split of Winter 1 and Winter 2 made them all comparable with each other with equal lengths S{Winter} = S {Summer}. States are defined in the text: J (juvenile/yearling), A (adult), M1 (migrant yearling), and M2 (migrant adult)

We used a multi-state mark-recapture (MSMR [28, 29];) model to estimate the survivorship of adults and yearling migrant and oversummering birds during winter (October – March) and summer (April – September). The four states are: J (juvenile or yearling); A (adult); M1(migrant yearling – unobservable state); and M2 (migrant adult – unobservable state), and the model also estimates the proportions of adults and juveniles that oversummer or migrate. A total of 1963 birds was captured, marked, and resighted in the analysis, which included data from 54 monthly ‘field campaigns’ conducted from October 2014 through March 2019. Birds marked prior to October 2014 were treated as having been marked when first resighted after 1 October 2014. Marked birds were subsequently resighted 5163 times, after multiple sightings within field campaigns were consolidated. Each of the 54 monthly field campaigns was assigned to one of five annual ‘sampling occasions’ (Winter 1, Winter 2, Spring, Summer, Fall; Fig. 2), for a total of 22 throughout the study. Repeat observations of the same individual during field campaigns within a sampling occasion were further consolidated, producing 3229 independent resighting records (Tables 4 and 5 in Appendix 1).

The structure of the model, with arrows indicating transitions, is shown in Fig. 2. Young-of-the year enter the model in Winter 1 as juveniles. Yearling birds transition to state A in Winter 1. All birds retain the stage assigned in Winter 1 when progressing to Winter 2. At the end of Winter 2, individuals either oversummer (remaining at Paracas), or migrate, in which case they transition to the unobservable states (M1 for yearlings, M2 for adults). The MSMR model estimates *ψ*^*JM*1^ (the probability that a yearling migrates), and derives *ψ*^*JJ*^ (the probability that a yearling oversummers) as its complement (1 - *ψ*^*JM*1^). Similarly, the model estimates *ψ*^*AM*2^ (the probability that an adult migrates), and derives *ψ*^*AA*^ (the probability that an adult oversummers) as its complement (1 – *ψ*^*AM*2^). Hence, *ψ*^*JM*1^, *and ψ*^*AM*2^ are the only transition probabilities estimated by the model. Spring, Summer and Fall are each 2 months long, and all birds retain their state with probability 1.0 as they progress through these successive stages. The cycle repeats beginning at Winter 1. Note that sampling occasions are not of equal length. Winter 1 (Oct, Nov, Dec) and Winter 2 (Jan, Feb, Mar) are 3 months long, while Spring (April, May), Summer (June, July) and Fall (August, Sept) are each 2 months long. Survivorship for the 6-month ‘summer’ is based on the Spring, Summer, and Fall sampling occasions, while the 6-month ‘winter season’ includes Winter1 and Winter 2 sampling occasions.

We competed a set of 36 versions of the basic model (Table 1), generated by combinations of 12 structures for annual survival (S), and three structures for the probability of resighting (p). There is a single structure for the transition probabilities. The model structures evaluating survival rates include all possible combinations of the one-way effects and two-way interactions, excepting the strategy*season interaction (impossible because strategies exist only in summer). The three-way interaction is not considered. Detection probability varies in three possible ways: by age, by season, or by age and season. We set the detection probability to zero for the unobservable states. Survival, detection and transition probabilities were constrained to be equal across the 5 years of observations, because models allowing annual variability failed to converge reliably.

**Table 1:** Set of models fitted for Semipalmated Sandpiper survival analysis. There are twelve structures for annual survival (S), three structures for probability of resighting (p), and a single structure for transition probability (***ψ***), not shown here. The 36 models are presented in ascending order by ΔAICc

Survival structure	Detection structure	N	AICc	Δ AICc	weight	Deviance
S(age + strategy+age*strategy)	p(age + season+age.season)	10	15,889.45	0.00	0.41	5554.57
S(age + strategy)	p(age + season+age.season)	9	15,891.20	1.74	0.17	5558.32
S(age + season+strategy+age*strategy)	p(age + season+age.season)	11	15,891.23	1.78	0.17	5554.34
S(age + season+strategy)	p(age + season+age.season)	10	15,892.54	3.08	0.09	5557.65
S(age + season+strategy+age*season+age*strategy)	p(age + season+age.season)	12	15,893.22	3.77	0.06	5554.31
S(strategy)	p(age + season+age.season)	8	15,894.13	4.68	0.04	5563.26
S(age + season+strategy+age*season)	p(age + season+age.season)	11	15,894.23	4.77	0.04	5557.33
S(season+strategy)	p(age + season+age.season)	9	15,894.93	5.48	0.03	5562.06
S(age)	p(age + season+age.season)	8	15,916.89	27.44	0.00	5586.02
S(age + season)	p(age + season+age.season)	9	15,918.79	29.33	0.00	5585.91
S(season)	p(age + season+age.season)	8	15,919.64	30.18	0.00	5588.76
S(age + strategy+age*strategy)	p(season)	9	15,920.33	30.87	0.00	5587.45
S(age + season+age*season)	p(age + season+age.season)	10	15,920.77	31.32	0.00	5585.88
S(age + season+strategy+age*strategy)	p(season)	10	15,922.14	32.68	0.00	5587.25
S(age + season+strategy+age*season+age*strategy)	p(season)	11	15,923.82	34.37	0.00	5586.92
S(age + strategy)	p(season)	8	15,926.58	37.13	0.00	5595.71
S(age + season+strategy)	p(season)	9	15,927.72	38.26	0.00	5594.84
S(age + season+strategy+age*season)	p(season)	10	15,928.41	38.95	0.00	5593.52
S(strategy)	p(season)	7	15,931.19	41.73	0.00	5602.32
S(season+strategy)	p(season)	8	15,931.55	42.10	0.00	5600.68
S(age + season+strategy+age*season+age*strategy)	p(age + season)	10	15,936.68	47.23	0.00	5601.79
S(age + strategy+age*strategy)	p(age + season)	8	15,937.69	48.24	0.00	5606.82
S(age + season+strategy+age*strategy)	p(age + season)	9	15,938.94	49.49	0.00	5606.06
S(age + season+strategy+age*season)	p(age + season)	9	15,939.24	49.79	0.00	5606.36
S(age + strategy)	p(age + season)	7	15,941.32	51.87	0.00	5612.45
S(age + season+strategy)	p(age + season)	8	15,942.31	52.86	0.00	5611.44
S(strategy)	p(age + season)	6	15,945.32	55.87	0.00	5618.46
S(season+strategy)	p(age + season)	7	15,945.72	56.27	0.00	5616.86
S(age)	p(season)	7	15,947.46	58.01	0.00	5618.59
S(age + season)	p(season)	8	15,949.38	59.93	0.00	5618.51
S(age + season+age*season)	p(season)	9	15,950.89	61.44	0.00	5618.01
S(season)	p(season)	7	15,951.94	62.49	0.00	5623.08
S(age)	p(age + season)	6	15,962.15	72.70	0.00	5635.29
S(age + season+age*season)	p(age + season)	8	15,963.11	73.66	0.00	5632.24
S(age + season)	p(age + season)	7	15,964.12	74.67	0.00	5635.25
S(season)	p(age + season)	6	15,965.74	76.29	0.00	5638.88

It is not possible to estimate unique survival rates for unobservable states in MSMR models [30], and hence it is typically necessary to set the survival probability of an unobservable state equal to that of one of the observable states. However, the combination of imposed constant annual survival and the structural determinism in transition probabilities, including the constraint that all individuals in unobservable states become observable in Winter 1 (Fig. 2), enables the estimation of age-specific survival probabilities for the unobservable states.

We fitted models in program MARK [31] using the “Rmark” interface [32] within program R, version 3.5.1 [33]. Model selection is based on Akaike’s information criterion, corrected for the effective sample size (AICc [34];). All models are used to estimate parameters and confidence intervals.

### Goodness of fit

The goodness-of-fit (GOF) tests available for MSMR models assume time-varying survival and fully observable states [35, 36]. Neither of these conditions holds in our model, and we therefore could not conduct GOF tests.

### Survival as a function of migration distance

The breeding destination of any individual Semipalmated Sandpiper at Paracas is unknown, but there is a strong relationship between breeding location and mean culmen length [24, 27], with bills shorter in western Arctic (~ 11,000 km migration) than in eastern Arctic (~ 8000 km) breeding populations. We use culmen length as a proxy for migration distance, and calculate survival in relation to migration distance as follows.

The relationship between culmen length and annual survival was previously estimated for yearlings by adding culmen length as a covariate to the encounter history and using an open robust design multistate model [37]. This produced a non-significant slope (survival probability/mm of culmen) of − 0.0048. However, this slope estimate combines oversummering and migrant yearlings, which, as we hypothesize, may differ in survival. We can decompose the estimate and calculate culmen length-specific survivorship rates for migrants by recognizing that, for each culmen size class, the survival estimate is composed of the survival of migrants and non-migrants, weighted by their proportion of the population. Denoting survival in culmen length class *i* as *S*_*i*_*,* the proportions of migrants and oversummerers as *Pm*_*i*_ and *Po*_*i*_*,* and the survival of migrants and oversummerers as *Wm*_*i*_ *and Wo*_*i*_, then

1 $$ {S}_i=\left({Pm}_i\ast {Wm}_i\ \right)+\left({Po}_i\ast {Wo}_i\ \right) $$

The proportions of migrants and oversummering yearlings in size class *i* are estimated based on pre-migratory molt patterns ([18]; see Appendix 2). With the reasonable assumption that the survival of oversummering birds (*Wo*_*i*_) is independent of culmen length class, the only unknown parameter is the survival of migrant juveniles (*Wm*_*i*_ in Eq. 1). Solving for *Wm*_*i*_

$$ {\mathrm{Wm}}_{\mathrm{i}}=\left({\mathrm{Pm}}_{\mathrm{i}}+\left({\mathrm{Po}}_{\mathrm{i}}\ast {\mathrm{Wo}}_{\mathrm{i}}\right)\right)/{\mathrm{S}}_{\mathrm{i}} $$

We apply this procedure to estimate the survival of yearling migrants in each culmen size class. We are unable to perform a parallel analysis for adults because we lack a marker of their migratory status comparable to that provided by the pre-migratory molt patterns of yearlings.

### Predicting the oversummering survival advantage

Our prediction is that the behavioural decision to migrate or not depends on the extra survival gained by oversummering (the ‘survival advantage’) being large enough to offset the foregone reproduction. The estimation of this theoretical threshold value is based on a simple life history model (see Appendix 3) that expresses the foregone breeding opportunity as a proportion of expected lifetime reproductive success. The predicted thresholds are 0.134 for yearlings, and 0.240 for adults. Individuals should migrate when their expected reproductive return exceeds this value, or oversummer when their expected return is lower.

## Results

### Survival estimates

The 1963 marked Semipalmated Sandpipers were resighted 3229 times within sampling occasions after marking. The percentage of birds not seen after first capture was on average 43% (annual range 31–55%). Marked birds were re-encountered on 1 to 15 subsequent sampling occasions (mean 1.64). The mean number of years a marked bird was re-encountered subsequent to initial capture averaged 0.85, ranging from 0.40 to 1.14 annually (excluding the logical zero from the final year). Further detail on the distribution of encounter periods is given in Appendix 1.

**Fig. 3:**
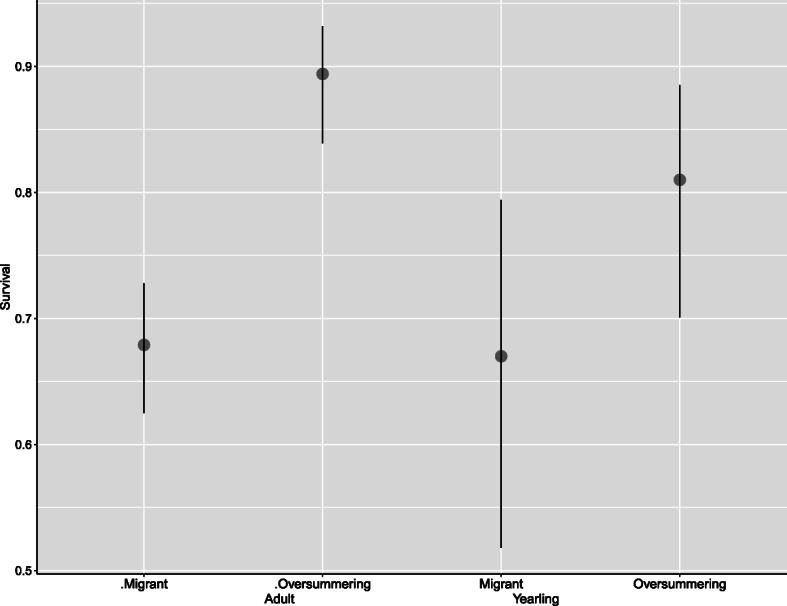
Seasonal (six-month) survivorship estimates of migrant and oversummering Semipalmated Sandpiper adults (left) and yearlings (right). Vertical lines are 95% confidence intervals

The model competition is summarized in Table 1. In the most informative model, survival varies by age and strategy, and by their interaction: juveniles have lower survival than adults, and migrants have lower survival than oversummering birds. The age by strategy interaction arises because the age difference in survival is non-existent for migrants, as shown by the survival estimates given in Table 3. Adult survival exceeds that of yearlings in both winter (adult 0.904; yearling 0.829) and summer (adult 0.894; yearling 0.810) by about 8%, but the survival of migrant adults and yearlings does not differ (adult 0.679; yearling 0.670) (Fig. 3).

Detection probability in all the most informative models varies by the age, season and their interaction, ranges from 0.171 to 0.594, and is higher during winter than summer for both age classes. During the summer period, adults have a higher detection probability than juveniles (Table 2).

**Table 2:** Detection probabilities (model averaged) of juveniles and adults in the (six-month) Winter (October to March) and Summer (April to September) seasons UCL/LCL Upper/Lower Confidence Limits (95%)

STATE	Probability	LCL95%	UCL95%
**J winter**	0.594	0.489	0.691
**J summer**	0.171	0.171	0.268
**A winter**	0.444	0.424	0.464
**A summer**	0.390	0.372	0.409

The top model carries 41% of model weight, and is 1.74 AICc units better than the next best model (Table 1). The second most informative model is identical in structure but excludes the interaction, carries 17.1% of the weight, and is 0.04 AICc units better than the third most informative model. This model includes the age, season and strategy effects, and the age by season interaction. It carries 16.8% of the weight. Seventy-five percent of the weight is in the top three models, which are all very similar.

We estimated annual survival as the product of the appropriate seasonal estimates in Table 3, namely (winter*summer) for oversummering birds, and (winter*migration) for migrants. The annual survival (Oct – Sept) estimated by this method is, for migrant yearlings 0.555, for oversummering yearlings 0.671, for adult migrants 0.614, and for oversummering adults 0.808.

**Table 3:** Seasonal (six-month) survival estimates (model averaged) for juvenile/yearlings (J) and adults (A) during Winter (October to March), Summer (April to September) and migration seasons. UCL/LCL = Upper/Lower 95% Confidence Limits

State	Survival estimate	95%LCI	95%UCL
**J winter**	0.829	0.643	0.929
**J summer**	0.810	0.701	0.885
**A winter**	0.904	0.838	0.945
**A summer**	0.894	0.839	0.932
**J migrant**	0.670	0.518	0.794
**A migrant**	0.679	0.625	0.728

### Oversummering

The probability that a yearling migrates (transition probability *ψ*^*JM*1^) is estimated at 0.72 (LCL: 0.67; UCL: 0.77), while the probability that an adult migrates (transition probability *ψ*^*AM*2^) is estimated at 0.81 (LCL: 0.79; UCL: 0.82). The (complementary) rates of oversummering are 0.28 (yearling) and 0.19 (adult).

Our main prediction is that oversummering gives a survival advantage large enough to offset the reproduction necessarily foregone by oversummering. For yearlings, the difference between the estimated survival (see Table 3) of migrants (0.670) and oversummering individuals (0.810) is 0.140 (95% CI 0.118–0.162). For adults, the difference between the estimated survival of migrants (0.679) and oversummering individuals (0.894) is 0.215 (95% CI 0.169–.261). Thus, the estimated survival advantages for adults and yearlings both closely match and do not differ significantly from the threshold values predicted (adults 0.240; yearlings 0.134; see Appendix 3).

### Survival in relation to migration distance

Based on pre-migratory molt patterns measured at Paracas [18], the probability of migration rises with culmen length in all years (Appendix 2), demonstrating that longer-billed (shorter migration distance) yearlings are more likely to migrate. We entered migration probabilities based on this relationship into Eq. 1 to estimate summer season survivorship in each culmen class length. As a sensitivity analysis, we varied summer season survival of oversummering yearlings between our estimate of 0.81 and the higher estimate of 0.93 for yearling Western Sandpipers oversummering at Paracas [37].

**Fig. 4:**
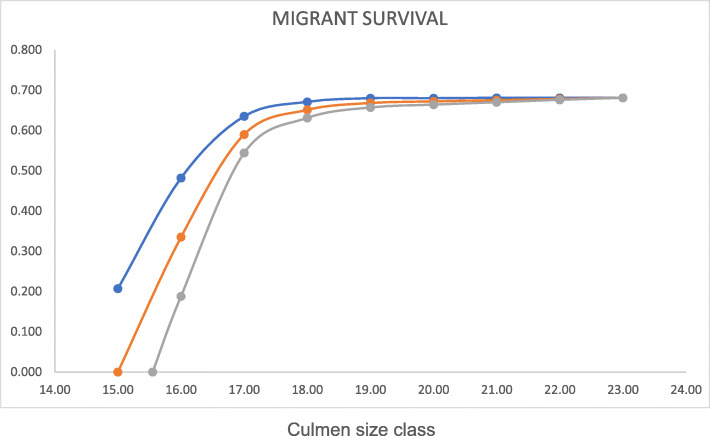
Calculated survival of migrant yearling Semipalmated Sandpipers at Paracas, in relation to culmen length (mm). Method described in the text. Longer culmens are associated with eastern breeding populations and shorter migration distance. Oversummering survival adjusted to 0.81 (upper line), 0.87 (middle line), or 0.93 (lower line)

Results are presented in Fig. 4. In all cases, the calculated survival of migrants falls off steeply for the short culmen classes (presumed to be western Arctic breeders with longer migration distance), but is level for longer-billed, shorter-distance migrant birds presumed to be from central and eastern Arctic breeding sites.

## Discussion

Of the several thousand Semipalmated Sandpipers that spend the non-breeding season at Paracas, Perú, an estimated 72% of yearlings and 81% of adults migrate northward to breed, with the remainder oversummering. We estimate that the summer (April – September) survival probability of oversummering yearlings is 0.140 higher than that of migrant yearlings, while that of oversummering adults is 0.215 higher than that of migrant adults. These estimates are statistically indistinguishable from the values theoretically required to compensate oversummering birds for their foregone expected breeding success. These results support the hypothesis that oversummering is a life history tactic undertaken when the survival advantage gained equals or outweighs in fitness terms the lost breeding opportunity. We assume that individual decisions to migrate or not are flexible, dependent on individual situations, including probable migration distance, and governed by mechanisms evolved by natural selection. Since our study is observational, we could not assign individual birds to oversummering or migratory strategies at random, as would be done in a true experimental study. Thus, the comparison is imperfect as a quantification of the consequences for individuals of migration vs. oversummering. There also may be biases in permanent emigration. Despite these caveats, we view these results as demonstrating the adjustment of a behavioural threshold at fitness equivalency between conditional alternative tactics of oversummering and migration, driven by the substantial survivorship advantage for oversummering birds of both ages.

It has long been hypothesized that oversummering provides a survival advantage over migration [17]. Though consistent with the hypothesis, previous comparisons of survivorship are confounded either by age or by location. For example, in Western Sandpipers at Paracas, oversummering yearlings have higher survival (0.83) than migrant adults (0.70 [37];). But this comparison is confounded by age because at this location all adults migrate and all yearlings oversummer [22]. Survivorship comparisons have also been made between overwintering groups at locations where (some) individuals oversummer (e.g. yearling male Western Sandpipers in Mexico; survival 0.65) and those where all oversummer (Chitré; survival 0.83) but this comparison is confounded by location. Reneerkens et al. [38] found that the apparent annual survival of Sanderlings wintering in tropical West Africa (Mauritania: 0.74 and Ghana: 0.75) was lower than at three European sites (0.84, 0.84 and 0.87), even though those from the tropics often oversummered. These estimates pool migrant and oversummering birds within locations, and so if the result from our study applies, would underestimate the survival of oversummering birds and overestimate that of migrants.

Oversummering is a form of ‘partial migration’, though reversed from the usual system in that migrants leave non-breeding rather than breeding areas. Buchan et al. [39] review published comparisons in partial migration systems of the survival of migrants and non-migrants, in birds and other taxa. They assembled 129 effect size estimates from 23 studies, of which 73% report a survival advantage for residents (i.e. non-migrants), 22% for migrants, and 5% report equal survival. The ‘persistently higher’ fitness advantage in birds is associated with survival, and not breeding success.

Our finding that survival falls with migration distance, although anticipated, comes with a number of caveats that must be borne in mind. First, the breeding location of any individual bird observed at Paracas is not known, but inferred from the association between the mean culmen length and breeding location. The correlation between migration distance and culmen length is therefore indirect. With the variation around population averages, a bird with the overall mean culmen length of 18 mm, although most likely to breed in the centre of the range, could possibly breed at any location.

The overall proportion of yearling migrants (72%) was estimated by our MSMR model, and we estimated the proportion of migrants within each culmen size class based on the incidence of partial post-juvenal wing (PPW) molt. This is a minimum estimate, because it has been established [40] that some individuals migrate without PPW, though the number of recaptures is too small to establish a reliable estimate of its frequency. The data are clear that PPW at Paracas occurs with higher frequency among long-billed birds, but the quantitative relationship of culmen length to migratory tendency retains some uncertainty. Our model estimates that 19% of adults oversummered, which is higher than we expected. Accounts of oversummering in the shorebird literature refer almost exclusively to young birds. The sole published measure of adult incidence of which we are aware is 8% of Semipalmated Sandpipers from Brazil [41];). Based on the migration distance pattern we documented for juveniles, the high proportion more likely relates to Paracas lying on the southern edge of the species’ wintering range. Nevertheless, the paucity of previous descriptions is curious.

We calculated that migratory survival is higher for long-billed (short-distance, eastern-breeding) migrant yearlings than for short-billed (long-distance, western-breeding) birds, consistent with the migration distance hypothesis. The calculated relationship (Fig. 4) is not linear: survival seems high and steady for bird with culmens longer than ~ 19 mm, and falls off quickly below that length. Though consistent with our hypothesis, we emphasize that this result must be viewed as tentative.

The life history model (Appendix 3) calculates the threshold survival advantage required for oversummering to match the reproductive cost of a missed breeding season. Oversummering is favoured at values above the threshold, and migration below. The survival advantages estimated from the data (adults 0.215, for yearlings 0.140) are statistically indistinguishable from the calculated threshold values of s* (for adults 0.240, for yearlings 0.134). Substantial numbers of both adults (19%) and yearlings (28%) oversummer, and we therefore presume that migration is flexible. We hypothesize that the migration decision is condition-dependent: individuals evaluate based on their own condition and circumstances whether they lie above or below the threshold. Under this hypothesis, those that migrate (the majority: 81% of adults and 72% of yearlings) decided that their migratory prospects were good enough that the extra survival that would be gained by oversummering is less than the threshold. The minority that oversummer, in contrast, decided that their migratory prospects were poor enough that the extra survival that would be gained by oversummering lies above the threshold. Factors contributing to the variance around our measured estimates of the thresholds likely include annual differences in the food availability at Paracas that supports migratory preparation (e.g. due to ENSO values [42]), differences in the proportions of birds from different breeding sites (because migratory distance has a strong influence on oversummering), the frequency distribution of pre-migratory condition in the overwinter population, and the accuracy with which individuals are able to assess their own condition.

Our data indicate that migration distance is an important consideration affecting migratory survival, with longer migration making oversummering more advantageous. Supporting this, a recent study from Martinez-Curci et al. (2020 [43];) showed a higher percentage of oversummering yearlings (53%) and adults (46%) Red Knots at a very distant non-breeding site in Argentina. Additional rigors such as long ocean crossings or predators may amplify the effect of distance. Physiological condition likely also bears on the decision, including factors previously suggested, such as health [9–11], or plumage condition [13]. Foraging ability and food conditions [12], such as those engendered by ENSO events or other ecological conditions, may also play an important role in the evaluation of whether a migration is worth the risk [39].

The data show that adults have higher survival than juvenile/yearlings, whether measured on an annual basis, or by seasons. Age differences of this kind have often been reported, so this is not unexpected. Among shorebirds, young birds are disadvantaged through foraging competition with adults, and so are expected to have poorer survival. For example, juvenile Red Knots *Calidris canutus canutus* at Mauritania are displaced by adults in dyadic interactions and are forced to use more dangerous feeding areas [44]. Wintering juvenile Redshanks *Tringa totanus* on a Scottish estuary are socially constrained by adults to feed on salt marshes, where higher exposure to raptors elevates the mortality rate [45]. But our seasonal comparisons reveal an interesting wrinkle, in that the survival difference between migrant adults and yearlings is non-existent.

The lower survival of young birds is often attributed to lack of experience in coping with migration, foraging and predators [46–49]. For example, juveniles are assumed to be naive about avoiding dangerous sites [50], or to be less capable than adults at finding good habitats [51, 52]. Our survival estimates show that yearlings and adults differ little or not all in migratory survival, which suggests little influence of competition or inexperience in this phase of the annual cycle. Note however that juveniles on their initial southward migration are not represented in our model.

## Conclusion

Our results support the life history hypothesis that both oversummering juvenile and adult birds compensate for the loss of a breeding opportunity with higher survivorship than migrant birds. Migration distance has been previously identified as a factor associated with migratory propensity, and our data support this conclusion. Other factors are likely also important in affecting the decision to oversummer. The Semipalmated Sandpipers studied at Paracas may be particularly sensitive to changes in other factors, since both strategies are currently maintained in the population. Factors affecting pre-migratory body condition, such as El Niño may affect the annual trade-off [53], and climate change could alter the balance over the longer term. Heightened migratory danger from increasing falcon populations [14–16] could also do so.

## Figures and Tables

**Fig. 5:**
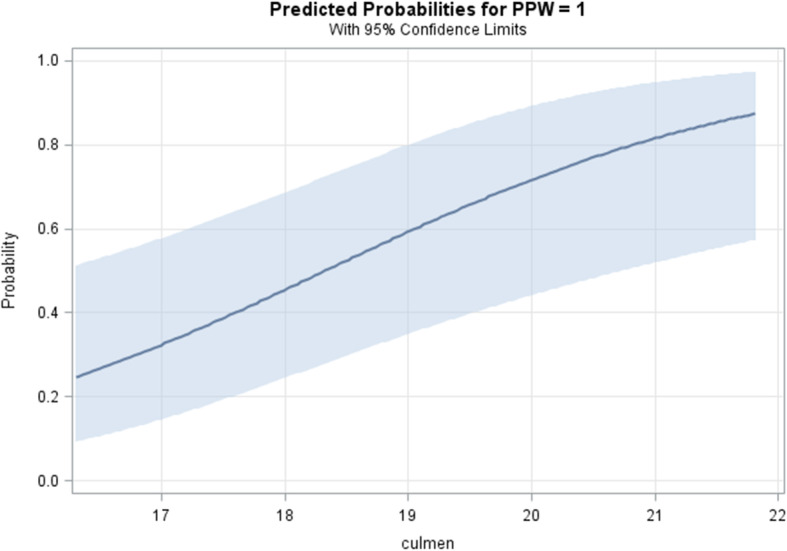
Probability of Partial Post-Juvenal Wing Molt (PPW) in juvenile Semipalmated Sandpipers captured at Paracas, during the 2015 pre-migratory season [18]. The relationship is computed with the co-variate day-of-year set to 168.1, breeding plumage index set to 1.54, and mass to 24.63 g, values that minimize bias. The probability of PPW ranges from 0.22 for the shortest bills to 0.83 for the longest

## Data Availability

The datasets used and/or analysed during the current study are available from the corresponding author on reasonable request.

## Appendix 1

### Encounters of marked Semipalmated Sandpipers at Paracas, Perú

**Table 4:** The number of sampling occasions in which birds marked in each year were (re-) encountered, ranging from 1 (= initial capture only), to 16 (includes initial capture). A total of 1963 were marked, of which 855 (43.6%) were encountered only during the sampling period when intially captured

Year	1	2	3	4	5	6	7	8	9	10	11	12	13	14	15	16	Total
**2014**	309	247	119	96	71	48	20	24	19	18	6	9	4	2	1	1	994
**2015**	196	100	54	32	20	16	7	10	4	4	2	.	.	.	.	.	445
**2016**	117	38	25	11	7	5	5	1	1	.	.	.	.	.	.	.	210
**2017**	68	38	14	12	6	7	4	.	.	.	.	.	.	.	.	.	149
**2018**	165	.	.	.	.	.	.	.	.	.	.	.	.	.	.	.	165
**Total**	855	423	212	151	104	76	36	35	24	22	8	9	4	2	1	1	1963

**Table 5:** Mean number of sampling occasions and mean number of years in which individually-marked Semipalmated Sandpipers were subsequently re-encountered at Paracas, Perú, both by year birds were initially marked

	No. sampling occasions			Number of years			
	Mean	SD	Range	Mean	SD	Range	N
**2014**	2.17	2.62	1–15	1.14	1.23	1–5	994
**2015**	1.49	2.04	1–10	0.82	0.98	1–4	445
**2016**	1.04	1.61	1–8	0.54	0.83	1–3	210
**2017**	1.24	1.62	1–6	0.40	0.88	1–2	149
**2018**	0	0	0	0	–	–	165
**overall**	1.64	2.31	15	0.85	1.09	1–5	1963

## Appendix 2

### Estimating relationships between culmen length, the probability of migration and survival

Tavera (2020 [37];) analyzed the relationship between culmen length and annual survival by adding culmen length as a covariate to the encounter history of marked Semipalmated Sandpipers at Paracas, using an open robust design multistate model to estimate survival. The resultant slope (− 0.0048; change in survival probability/mm of culmen) is slightly but non-significantly negative, which runs counter to the expectation for migrants. However, this slope estimate pools oversummering and migrant yearlings, which we hypothesize differ in survival.

The procedure to make separate survival estimates for migrants and oversummering birds is described in the text, and is encapsulated in eq. 1. This requires information on the proportion on migrants in each culmen size class, derived as follows. Tavera et al. ([37]; see her Table 7) measured the proportion of yearlings undergoing ‘partial primary wing moult’ (PPW) at Paracas during the pre-migratory period. Three years of data all show higher rates of PPW at longer culmen lengths, with the strongest relationship and the narrowest confidence limits occurring in 2015 when PPW was most common (44.4%), shown below (Fig. 5 in Appendix 2). Gratto and Morrison [40] observed that some yearlings may migrate without any PPW, and as no individuals undergo PPW and then oversummer (Tavera unpubl. data), the incidence of PPW appears to be a minimal estimate of the proportion of migrants.

To estimate the proportion of migrants in each culmen size class, we adjusted the estimate of PPW in each size class upward, requiring that its incidence increases smoothly (i.e. no inflection points) over all culmen length size classes. Values were adjusted until (i) the overall proportion of migrants and (ii) the overall survival match the values estimated by the MSMR model (0.72 and 0.70, respectively). The final vector of proportions (‘prop. Migrants’ column in Table 6 in Appendix 2) is not a unique solution, but with the known distribution of culmen lengths at Paracas (‘proportion’ column in Table 6 in Appendix 2, from Fig. 2 in [18]), only a narrow range of size class proportions is able to satisfy these criteria. These estimates were entered into Eq. 1. The calculations are summarized in Table 6 in Appendix 2.

**Table 6:** Calculations of the survival of migrant Semipalmated Sandpipers at Paracas in each culmen length size class. For example, survival in the 17 mm culmen length size class is 0.710. These constitute 23% of the birds at Paracas. We estimate using the above procedure that 57% are migrants, so 43% oversummer. Oversummer survival is 0.81. Therefore, if overall survival of the size class is 0.710, migrant survival is 0.635, and oversummering gives a survival advantage of 18%

Culmen (mm)	Proportion	Survival	Prop. (migrants)	Prop. (oversummer)	Migrant survival	Survival advantage
15.0	0.01	0.720	0.15	0.85	0.207	0.60
16.0	0.06	0.715	0.29	0.71	0.482	0.33
17.0	0.23	0.710	0.57	0.43	0.635	0.18
18.0	0.31	0.705	0.75	0.25	0.671	0.14
19.0	0.23	0.701	0.84	0.16	0.680	0.13
20.0	0.09	0.696	0.88	0.12	0.680	0.13
21.0	0.05	0.691	0.92	0.08	0.681	0.13
22.0	0.01	0.686	0.96	0.04	0.681	0.13
23.0	0.01	0.681	1.00	0.00	0.681	0.13

## Appendix 3

### How large must the oversummering survival advantage be to compensate for a missed breeding season?

Several thousand Semipalmated Sandpipers spend the boreal winter at Paracas. Northward migration to breeding sites begins in April, but some yearlings (i.e. birds born the previous summer) as well as some adults do not migrate but remain at Paracas during the boreal summer (‘oversummer’).

A hypothesis for oversummering is that survival is higher than for migration. There is also a cost to oversummering, because a breeding opportunity is foregone. The survival advantage for oversummering must therefore be high enough, in fitness terms, to compensate. As implied by previous studies of shorebird oversummering (see Introduction), we hypothesize that the ability to undertake the breeding migration is condition-dependent. Due to relatively poor condition, (perhaps due to parasitic infection, low wing or plumage quality, or low fat stores [42]) some individuals decide to oversummer, trading off the fitness benefit of higher survival against the fitness cost of a foregone breeding opportunity. In this Appendix we estimate the magnitude of the survival advantage required to compensate.

We term the expected Lifetime Reproductive Success (LRS) of an adult *A*. We assume that this is independent of whether a bird migrated or oversummered as a yearling (i.e. no carry-over). The expected reproductive success of a breeding yearling is termed *R*, and is likely lower than that of adults. We use the value of 0.76 from Weiser et al. (2018 [54];) for the annual survival of adult semipalmated sandpipers. (This is slightly higher than that estimated at Paracas, where mortality and permanent emigration cannot be distinguished.) With this level of survival, an adult Semipalmated Sandpiper expects to survive for 1/(1–0.76) = 4.2 years.

Gratto et al. (1983 [6];) measured Semipalmated Sandpiper reproductive success: mean adult clutch size is 3.9 and hatching success 77%, while yearling clutch size is 3.8 and hatching success 44%. The expected annual reproductive success of adults is therefore 3.00, and of yearlings 1.67. The expected LRS of an adult *A* is (3.00 * 4.2) = 12.5.

We term the breeding season (April – August) survival of a migrant yearling *x,* and denote the additional survival gained by oversummering as *s* (i.e. the survival advantage). We seek the value of *s* at which the fitness of a yearling’s life history with oversummering is equal to that of a life history with migration. Designate this threshold value of *s* as *s**. Oversummering is favored if *s* > *s**, and migration if *s* < *s**.

To find *s**, we reason as follows: from the decision point (i.e. in March or April, when a bird commits to one strategy or the other) an oversummering yearling expects to survive until October and reach adulthood with probability *x + s*, and from that point expects LRS of *A*. Expected fitness is *(x + s)A*. From the same decision point, yearling migrants survive the breeding season and reach adulthood with probability *x*, and expect LRS of *A* if they do so. Reproductive success of the first breeding attempt is *R,* so expected fitness is R + *xA*. At the threshold these alternatives have equal fitness, so *(x + s)A = R + xA*. Solving for *s* gives *s* = R/A*. The term *R/A* can be interpreted as the proportion of expected adult LRS that a yearling foregoes by oversummering.

The predicted threshold survival advantage *s* = R/A* = (1.67/12.5) = 0.134. The threshold survival advantage required for adults to oversummer is predicted to be higher because they forego more reproduction by oversummering than do yearlings. Following the logic above we calculate *s** for adults as 0.240.

Note also that yearling migratory survival is estimated to be lower in smaller culmen size classes (Fig. 4) presumably because the migration distance is on average longer. This would enlarge the survival advantage of oversummering, assuming that all else is equal. We can estimate the survival advantage for each culmen size class by subtracting the estimated migrant survival of migrants in each culmen size class from the measured oversummer survival estimate of 0.81. Assuming that neither *R* nor *A* is affected by culmen length, the threshold value of *s** remains the same (0.13). The pattern of survival advantage therefore matches migratory propensity (estimated from PPW), with more oversummering in smaller culmen length classes (Table 6 in Appendix 2).

Finally, we add that this basic theoretical calculation of the threshold does not consider the option of PPW. We presume this would enlarge somewhat the conditions for migration (i.e. lower *s**) because it enables an improvement in migratory preparedness (specifically, the condition of primaries) at a cost lower than that of a full wing molt.
